# Selections

**Published:** 1892-08

**Authors:** 


*Secretary.*

---

### Selections.

### PHILADELPHIA COUNTY MEDICAL SOCIETY.

The following abstract is given from Dr. S. Solis-Cohen's paper, "Shall Physicians become Sales-Agents for Patent Medicines?"

"In an address to the Medical Society of the State of Pennsylvania at its last meeting, in calling attention to the alliance between the secular press and the empirics and nostrum-venders, I

felt justified in saying that the publishers of magazines and newspapers that allowed themselves to advertise the curative virtues of this or that ready-made preparation or alleged remedial measure, to be applied indiscriminately to all cases,—whether of one disease or of many diseases,—were accessories to a crime against the unfortunate and the helpless; not alone because of the money filched from the pockets of those deluded by the false promises held out to them; not alone because of suffering unrelieved and lives deprived of their chance for prolongation; but in many instances because of the disease and suffering and death directly produced by the poisonous compounds and noxious gases administered to any that chose to purchase. If such criticism were justified—and who is here that will deny its truth?—if such criticism were justified when applied to those who make no pretence of special knowledge or of devotion to a noble art,—to those whose object is solely and avowedly commercial,—what language remains to characterize the action of medical journals that permit the insertion in their columns of advertisements such as these that I pass around? What severity of reprobation is adequate for the conduct of the *Journal of the American Medical Association*? What words of condemnation are strong enough for the physician that permits his name to be associated with these devices of the devil?

"The frankly unscrupulous patent-medicine vender, the maker of 'Safe-cures,' or 'Temperance Bitters,' or 'Sure Specifics,' is at least to be commended for what, to paraphrase a remark of Senator Benjamin Harrison's, may be termed his 'bold brutality.' His allegations of philanthropic motives are not intended to be believed; they deceive no one—they are the recognized *ad captandum* devices of the cleverer advertiser—and, in the sale of his wares, there is no pretence of examination, or of diagnosis, or of prescription based on diagnosis.

"Far more iniquitous and far more dangerous to society is the wily manufacturer that advertises 'to the profession only.' Whether he ostentatiously holds secret the composition of his nostrum, or whether with pretended frankness he describes it with an appellative that means nothing, or publishes a formula that cannot be carried out, his object is the same; he seeks to make the physician's the hand whereby he may reach pockets shut from the coarser methods of the Warners, the Pinkhams, and the Jaynes; for, after all, it is the minority that can be deluded by the flaring posters of 'Wizard Oil,' or the lying testimonials of 'Tonic Vermifuge.' When a sick man applies to a physician, thinking that thereby he will secure the benefit of special knowledge brought to bear upon the conditions of the individual case, intrusting to the conscience of his medical adviser his health and his life, he is entitled to the skill and the thought for which he pays, and that he deems himself to be receiving. He certainly deserves better treatment than to be handed over to the mercies of 'antikamnia,' or 'febricide,' or 'quickine,' or 'gleditschine,' or 'Freligh's tablets,' or 'Listerine,' or any other of the unholy crew. If such is to be his fate, let him have the satisfaction of buying the worthless or poisonous stuff direct, without the sham of a professional consultation, and without paying purchaser's commission to the medical sales-agent.

"At the coming meeting of the State Society I propose offering the following resolutions, for which I ask the support of this Society:

"*Resolved*, That the Medical Society of the State of Pennsylvania hereby expresses its highest disapprobation of the practice of giving certificates or testimonials to secret preparations alleged to be of medicinal virtue, and calls the attention of the affiliated county societies to the fact that such action on the part of members of the said societies is in derogation of the dignity of the profession, and in violation of the letter and the spirit of the Code of Ethics of the American Medical Association and of this Society.

"*Resolved*, That this Society likewise expresses its disapprobation of the practice of inserting advertisements of secret preparations in the columns of medical journals, such action being an insult to the intelligence of the profession, and a degradation of journals indulging therein to the level of the patent-medicine almanac. Especially to be condemned is the action of the *Journal of the American Medical Association* in admitting such advertisements.

"*Resolved*, That copies of these resolutions, duly attested by the permanent secretary, be sent to all county societies in affiliation with this Society, to the American Medical Association, to State medical societies in affiliation therewith, and to the publishers and editors of American medical journals."

On motion of Dr. J. Madison Taylor, the resolutions were adopted as the sense of the Philadelphia County Medical Society, and the delegates to the Medical Society of Pennsylvania were instructed to officially present and support them.—*Journal of the American Medical Association.*

### ABSTRACTS FROM THE RESPONSE OF THE EDITOR OF THE "JOURNAL OF THE AMERICAN MEDICAL ASSOCIATION."

"To all questions there are two sides, and it seems that the reasons for the course which the *Journal* has pursued in this matter should be set before the members of the Association.

"The ideal in medical journalism has not, and never will be, attained, and it is well that this is so, for then the ambitious journalist would in a great measure cease his labors.

"Not infrequently a physician is seized with an ambition to show his fellows just what a medical journal should be and what it should do, and one of the first propositions in his mind is a publication that will be absolutely clean and free from all repugnant advertisements, and, in his estimation, all advertisements are repugnant.

"A theory is adopted that an announcement to the medical profession of a medical journal without advertisements will cause hundreds and thousands of names to be sent in as subscribers. The theory is excellent, the ideal is on a high plane; but, unfortunately, the hundreds of expected names do not come in, but, instead, the printer's monthly statement is singularly prompt, and, if not at once paid, a dun quickly follows, saying paper bill is due, and printers must have their wages every week, to say nothing of rent, taxes, etc.

"The luxury of furnishing copy and seeing a clean publication is highly enjoyed. English adjectives are not sufficiently strong to do justice to the publication; but the vexatious counter-irritation produced by the aforesaid bills fairly blisters the ideal journal, the monthly application soon becomes monotonous, and a running sore is established. The ambitious editor loses patience with all other members of his profession for not flocking to his relief, and as they don't flock, he is apt to indulge in adjectives and resolutions of condemnation. There is no hazard in saying there is not an editor in this country who would not greatly prefer the management of a journal without advertisements than one with them. In the founding of the *Journal of the American Medical Association*, the trustees appointed by that body would have much preferred to start the *Journal* without advertisements than with them; but the means at their disposal were not only small, but very limited, so that the question presented to them was not of an exclusion of advertisements, but one of founding a journal that would have sufficient life-giving properties to exist after its establishment.

"The line to be drawn by the publisher must always be guided by his own judgment, and a maturity of knowledge in this direction comes only with an experience of years, and may justly be classed with the skill of an expert in a professional specialty.

"For the growing use of such preparations several reasons may be assigned. Practitioners in the country and smaller communities do not have at their disposal the extensive pharmaceutical resources of their brethren in the larger cities, and to men so situated, compressed tablets, and articles of like description, have proven a great boon. The convenience of ready-made preparations once appreciated, the tendency of these gentlemen to lessen their already arduous labors by the further use of such preparations can be readily appreciated. They can, by their use, present their patrons with much more elegant specimens of pharmaceutical work than they can possibly produce from the crude and limited materials to be found in their saddle-bags. Probably these factors of convenience in dispensing and elegance in preparation have contributed more than all else to the use of these much-condemned proprietary preparations. Be that as it may, there certainly does exist on the part of the profession at large a wide-spread demand for just these things.

"That it is not scientific to use as remedies preparations whose composition is known but in part is certainly true, and that it is absolutely unjustifiable for any physician to use a preparation whose composition is entirely unknown is likewise true. Just what advertisements should be excluded from appearing in this particular publication is, of course, a matter of individual judgment, in which probably no two men would agree exactly.

"Exclude all advertisements of proprietary medicines, says the Philadelphia County Medical Society.

"To this we will not object if—the Philadelphia County Medical Society will donate to the *Journal* an equivalent sum of money, and furnish a guarantee that this will be continued for a long term of years; but even that could not well be afforded by the *Journal*, for the advertisements continuously furnish the members of the American Medical Association with information which many want, and which they could not otherwise obtain unless by taking some other journal.

"Furthermore, the art of pharmacy has kept fully abreast of the advances made in other departments of medicine. To such a laudable extent has the work of the pharmacists progressed, that the American Medical Association, two years ago, incorporated pharmacy with materia medica, and made of it a Section of the Association.

"The manufacturers of proprietary preparations should be required to make public the ingredients of such preparations. The method of manufacture and the skill used in preparation is their own property, and physicians need not have a practical knowledge of these methods in order to make an intelligent use of such remedies. A knowledge of the methods used for extracting quinine from cinchona bark is not needed by physicians any more than a knowledge of the method used in the manufacture of champagne before drinking, or of the baking a pudding before eating.

"Suppose the Philadelphia medical journals would all unite and throw out their advertising patronage, and at the same time double their subscription rate; how long would it take for the physicians of Philadelphia to say to their home journals, Erase our names; we can get an equally good, but no better journal, plus the advertising pages, published in New York for one-half the price you demand. We like to patronize home talent, but don't like to be robbed to pay for it. In that spirit and expression the Philadelphia physician would be but the counterpart of nine out of every ten the country over, and it is the subscriptions of the nine that sustain the journals.

"As rapidly as contracts expired during the past year, undesirable patrons of the advertising pages have been eliminated, and offers of orders during that time amounting to more than eighteen hundred dollars have been rejected because of their character. This work of elimination might, perhaps, have been carried further, but we can say without hesitation that the pages of the *Journal* are reasonably free from objectionable advertising matter, and that which is published represents to the Association for the current year a sum exceeding twelve thousand dollars.

"This amount is named, and easily verified, so that the Philadelphia County Medical Society may know approximately the sum annually needed to make good the losses from this source, and the cost of the luxury they have resolved upon.

"One other point. The advertising pages of this journal are completely divorced from the reading matter. No 'reading notices' appear in its pages. All matter which has any appearance of an advertising character about it is carefully excluded from its reading matter. The advertisements are understood to be paid material, *ex parte* statements, and to be taken for what they are worth, just exactly as advertisements are everywhere.

"In this connection, it is instructive to look at the advertising pages of the *British Medical Journal*, truly our sister, as it is published, like this journal, under the auspices of a great national medical association. Among the advertisements which appear in the last number which has reached us, we notice 'Bynin,' 'Bromidia,' 'Bovinine,' 'Cocatina,' 'Pepsalia,' 'Flitwick,' etc."

